# Immunoinformatics and Immunogenetics-Based Design of Immunogenic Peptides Vaccine against the Emerging Tick-Borne Encephalitis Virus (TBEV) and Its Validation through In Silico Cloning and Immune Simulation

**DOI:** 10.3390/vaccines9111210

**Published:** 2021-10-20

**Authors:** Muhammad Suleman, Muhammad Tahir ul Qamar, Kiran, Samreen Rasool, Aneela Rasool, Aqel Albutti, Noorah Alsowayeh, Ameen S. S. Alwashmi, Mohammad Abdullah Aljasir, Sajjad Ahmad, Zahid Hussain, Muhammad Rizwan, Syed Shujait Ali, Abbas Khan, Dong-Qing Wei

**Affiliations:** 1 Centre for Biotechnology and Microbiology, University of Swat, Swat 19200, Pakistan; suleman@uswat.edu.pk (M.S.); zahid@uswat.edu.pk (Z.H.); m.rizwan@uswat.edu.pk (M.R.); shujaitswati@uswat.edu.pk (S.S.A.); 2 College of Life Science and Technology, Guangxi University, Nanning 530004, China; m.tahirulqamar@hotmail.com; 3 Department of Plant Breeding and Genetics, University of Agriculture, Faisalabad 38000, Pakistan; kiranzahra9999@gmail.com; 4 Department of Biochemistry, Government College University, Lahore 54000, Pakistan; samaruaf@yahoo.com; 5 Department of Botany, University of Okara, Okara 56300, Pakistan; aneelarasool3@gmail.com; 6 Department of Medical Biotechnology, College of Applied Medical Sciences, Qassim University, Buraydah 51452, Saudi Arabia; 7 Department of Biology, College of Education, Majmaah University, Al Majma’ah 15341, Saudi Arabia; n.alsowayeh@mu.edu.sa; 8 Department of Medical Laboratories, College of Applied Medical Sciences, Qassim University, Buraydah 51452, Saudi Arabia; aswshmy@qu.edu.sa (A.S.S.A.); mjasr@qu.edu.sa (M.A.A.); 9 Department of Health and Biological Sciences, Abasyn University, Peshawar 25120, Pakistan; sahmad@bs.qau.edu.pk; 10 Department of Bioinformatics and Biological Statistics, School of Life Sciences and Biotechnology, Shanghai Jiao Tong University, Shanghai 200240, China; abbaskhan@sjtu.edu.cn; 11 State Key Laboratory of Microbial Metabolism, Shanghai-Islamabad-Belgrade Joint Innovation Center on Antibacterial Resistances, Joint Laboratory of International Cooperation in Metabolic and Developmental Sciences, Ministry of Education and School of Life Sciences and Biotechnology, Shanghai Jiao Tong University, Shanghai 200030, China; 12 Peng Cheng Laboratory, Vanke Cloud City Phase I Building 8, Xili Street, Nashan District, Shenzhen 518055, China

**Keywords:** Tick-borne encephalitis (TBE), proteome mining, vaccine construct, in silico cloning, immune simulation

## Abstract

Tick-borne encephalitis virus (TBEV), belonging to the Flaviviridae family, is transmitted to humans via infected tick bites, leading to serious neurological complications and, in some cases, death. The available vaccines against the TBEV are reported to have low immunogenicity and are associated with adverse effects like swelling, redness and fever. Moreover, these vaccines are whole-organism-based, carry a risk of reactivation and potential for significant mortality. Consequently, to design a potential antigenic and non-allergenic multi-epitope subunit vaccine against the TBEV, we used an immunoinformatic approach to screen the Tick-borne virus proteome for highly antigenic CTL, HTL and B cell epitopes. The proper folding of the constructed vaccine was validated by a molecular dynamic simulation. Additionally, the molecular docking and binding free energy (−87.50 kcal/mol) further confirmed the strong binding affinity of the constructed vaccine with TLR-4. The vaccine exhibited a CAI value of 0.93 and a GC content of 49%, showing a high expression capability in *E coli*. Moreover, the analysis of immune simulation demonstrated robust immune responses against the injected vaccine and clearance of the antigen with time. In conclusion, our vaccine candidate shows promise for both in vitro and in vivo analyses due to its high immunogenicity, non-allergenicity and stable interaction with the human TLR-4 receptor.

## 1. Introduction

The arboviruses (arthropod-borne viruses) of the genus *flavivirus* (family *Flaviviridae*) are transmitted to vertebrates through infected mosquitoes and ticks. These mosquito-borne and tick-borne flaviviruses are the major cause of jaundice, hemorrhagic disease, fever, rashes, and encephalitis in human beings [1]. Tick-borne encephalitis virus (TBEV), a member of tick-borne encephalitis virus complex endemic in Eurasia, is transmitted by ticks (ixodid ticks) and causes a neurological disease known as tick-borne encephalitis (TBE) [2]. During a biphasic course, the disease symptoms are initially similar to influenza, followed by a symptom-free phase, and finally, the neurological disease occurs [3]. Meningitis is a mild form of the disease, whereas the severe form of the disease, encephalitis, may lead to spinal cord paralysis, myelitis, and death [4]. In Eurasia, more than 10,000 infection cases are reported annually due to TBEV [5,6].

Phylogenetic reconstructions based on E protein data identified Siberian (TBEV-Sib), Far-Eastern (TBEV-FE), and European (TBEV-Eu) subtypes of TBEV. Furthermore, recent investigations also favor the recognition of the Himalayan (TBEV-Him) and Baikalian (TBEV-Bkl) subtypes in TBEV [5,7]. The mortality rates of TBEV-Sib, TBEV-FE, and TBEV-Eu are 6–8%, 40%, and 1–2%, respectively, depending on the patient’s age and immunity [8,9].

The virus is a positive single-stranded RNA (+ssRNA) of 11 kb, packed into an envelope having a diameter of around 50 nm [10]. The genome encodes a polyprotein that is processed co- and post-transcriptionally into three structural proteins, i.e., envelope glycoprotein (E), pre-membrane (preM) and capsid (C), and seven non-structural proteins (NS1, NS2a, NS2b, NS3, NS4a, NS4b, and NS5) [11]. Secondary structures of the 5′ and 3′ UTRs have an essential role in the life cycle of the virus [12,13].

To date, several licensed vaccines are available, which are based on different strains of Far-Eastern and European TBEV subtype. These vaccines can be categorized as Russian, European and Chinese [14]. Two European vaccines, German isolate K23 (Encepur) and Austrian isolate Neudoerfl (FSME-IMMUN) are currently available and both contain TBEV-Eu strains [15]. The Russian licensed TBEV vaccines are based on Far-Eastern strain 205 (EnceVir) and TBEV-FE isolates Sofjin (TBE vaccine Moscow and Tick-E-Vac/Klesch-E-Vac). In China, TBEV-FE strain Sen-Zhang is used in the approved SenTaiBao vaccine [16]. It has been reported that these vaccines cause mild to moderate local and systemic adverse effects, such as headache, fever, redness and swelling. Moreover, as they are whole organism-based vaccines, the viruses may, rarely, be reactivated and cause meningoencephalitis with substantial mortality [10]. The immunogenicity of these vaccines is also low. Considering this, new safe, broad-spectrum effective, and targeted vaccines are needed to support the above-mentioned vaccine in eradicating TBEV.

Recent advancements and great strides in bioinformatics have generated diverse in-silico approaches for the identification of novel vaccine and drug targets [17]. These in-silico approaches were helpful in developing cost-effective vaccines and drugs while reducing the time span [18]. Diverse in-silico studies were conducted over the last 10 years for the identification of mutations, novel vaccine candidates, and drug targets for controlling various pathogens such as *Porphyromonas gingivalis* [17], *Bartenolla bacilliformis* [19], *Norovirus* [18], *Mayaro virus* [20], *Epstein-Barr virus* [21], *Mycobacterium tuberculosis* [22,23], Staphylococcus aureus [24], *SARS-CoV2* [25,26] and *Streptococcus pneumonia* [27]. Immunoinformatics approaches are the most effective tools for the development of specific, stable, and multi-epitope vaccines. Immunoinformatics approaches are reliable, precise, and speedy, and therefore, this study was designed to develop a multi-epitopes subunit vaccine against TBE. For this purpose, antigenic proteins of TBEV were used for the prediction of CTL and HTL epitopes. The final vaccine construct was generated by combining the selected CTL and HTL epitopes. In silico cloning and immune simulation confirmed the therapeutic potential of the designed candidate vaccine.

## 2. Materials and Methods

### 2.1. Data Retrieval

**Figure 1:**
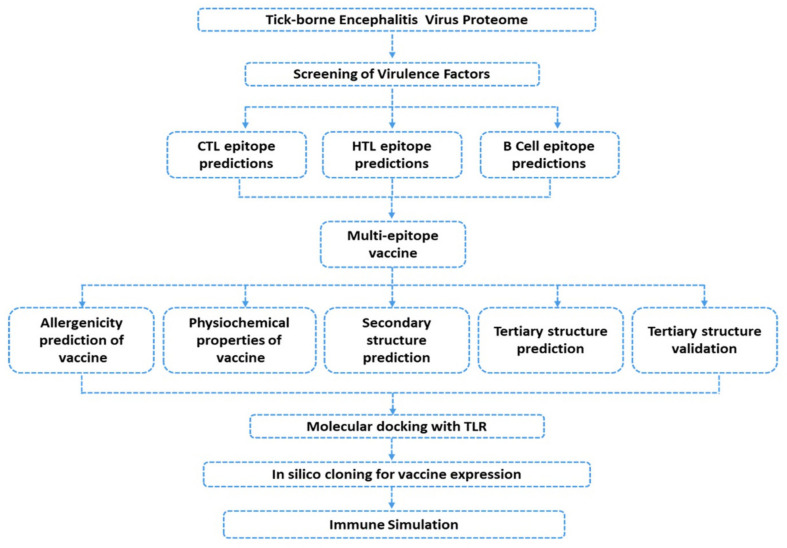
Hierarchical flow of the whole work. Antigenic factors were screened for the CTL, HTL, and B-Cell epitopes prediction. The final vaccine construct was obtained and checked for various properties. In silico cloning and expression was performed to check the efficacy of the final construct.

The study was started by fetching the complete proteome of tick-borne encephalitis (U27495) from Gene Bank Database of NCBI (https://www.ncbi.nlm.nih.gov/, accessed on 25 November 2020) [28] available under accession number: U27495. The stepwise flow of the study is illustrated in Figure 1.

### 2.2. Data Processing

#### 2.2.1. Prediction of Immunogenic Epitopes

CTL epitopes were identified in the polyprotein sequence using NetCTL 1.2, available at http://www.cbs.dtu.dk/services/NetCTL/ (accessed on 25 November 2020) [29]. This prediction combines three elements: first, it performs prediction for the MHC-I binding peptide, followed by C-terminal proteasomal cleavage, and lastly, executing the transportation efficiency Transporter Associated with Antigen Processing (TAP) program. The first two parameters were estimated via artificial neural networks, whereas TAP transporter efficacy was calculated through the weight matrix. The cut-off value used for CLT epitopes prediction was allowed at 0.75. Furthermore, HTL epitopes of 15-mer amino acids length were predicted showing a good affinity for human alleles: HLA-DRB1*01:02, HLA-DRB1*01:01, HLA-DRB1*01:04, HLA-DRB1*01:03, HLA-DRB1*01:05 using IEDB server at http://www.iedb.org/ (accessed on 25 November 2020) [30]. The predicted peptides were sorted based on an IC50 score and were grouped as: IC50 value < 50 nM (good binders), IC50 score < 500 nM (intermediate binders) and <5000 nM (low affinity binders). The percentile ranking was inversely proportional to the epitopes’ binding affinity, implying that a lower percentile rank is the depiction of higher binding affinity. To trigger the protective host antibody response, B cell epitopes were predicted using BCPred webserver. To predict Linear B cell epitopes in the virus polypeptide, an online web tool of BCPred was used [31]. To filter the best predicted B cell epitopes, a cut-off score of 0.8 was defined in the process. ElliPro [30] was further utilized to predict conformational B-cell epitopes. The ranking was based on the protrusion index (PI) score, which was assigned to each predicted epitope.

#### 2.2.2. Multiple-Epitope Vaccine Designing and Evaluation

Predicted epitopes prioritized in the previous steps were fused carefully to achieve a multi-epitope peptide vaccine construct. For this, CTL, HTL, and B cell epitopes were linked via AAY, GPGPG, and KK linkers, respectively [32]. Then, adjuvant was added at the N terminal of the vaccine sequence [33]. Allergenicity of the vaccine sequence was determined using a well reputed AlgPred server. This server can be accessed online at http://www.imtech.res.in/raghava/algpred/ (accessed on 25 November 2020) [34] and predicts allergic sequences at an accuracy of around 85%. Allergenic sequence can be identified if it has a score greater than threshold (>0.4).

#### 2.2.3. Prediction of Vaccine Antigenicity

The vaccine construct needed to be antigenic for eliciting the proper immune response. VaxiJen server was employed to predict antigenicity of the vaccine keeping the threshold at default 0.4 [35]. VaxiJen uses a new alignment-free approach for antigen prediction, which is based on auto-cross covariance (ACC) transformation of protein sequences into uniform vectors of principal amino acid properties. Additionally, allergenicity was predicted with the help of AlgPred server (http://crdd.osdd.net/raghava/algpred/ accessed on 25 November 2020) at an accuracy of around 85% [34] to check whether or not the vaccine construct will trigger the autoimmune reaction. AlgPred uses a consensus approach for the prediction of allergenicity scores. Allergenic sequence can be identified when there is a score greater than the threshold (>0.4).

Different physicochemical properties of the vaccine such as amino acid composition, molecular weight, theoretical pI, in vivo and in vitro half-life, instability index, aliphatic index, and grand average of hydropathicity (GRAVY) were unveiled for the vaccine through online ProtParam [36]. Secondary structure of the vaccine was predicted using a freely available online tool of PSIPRED [37].

#### 2.2.4. Multiple-Epitope Vaccine 3D Structure Prediction and Validation

For tertiary structure prediction, RaptorX (http://raptorx.uchicago.edu/ accessed on 25 November 2020) was utilized [38]. RaptorX also allows the prediction of secondary structure elements, solvent accessibility, and binding regions in the input sequence. The server also uses the confidence parameter to assess the quality of the predicted 3D structures. The quality of the models was examined using *p*-value, which is a global quality indicator, along with a global distance test (GDT) and un-normalized GDT (uGDT). Vaccine 3D structure was subjected to refinement using Galaxy Refine [39] for the purpose of improving local and global structure quality of the vaccine. During the process, the protein side-chain was reconstructed and repacked by employing CASP10 refinement and then relaxed through MD simulation. Quality of the vaccine 3D model was tested using online tools such as ERRAT and ProSA-web. The latter is commonly used in protein structure validation to investigate input 3D model quality [40], which is assessed by means of a quality score. In cases when the score of the input structure is within the score’s range of experimentally determined structure, the question structure most likely has no errors. The quality of the predicted 3D structure can be estimated by using the ProSA-web server, thus making it easy for users to improve the overall structure quality. ERRAT [41] is mainly deployed to find and fix non-bonded interactions present in the given structure.

#### 2.2.5. Ensemble Vaccine Folding Evaluation through MD Simulation

Proper folding of protein ensembles is required for cellular protein network regulation which plays different biological functions, i.e., immune response triggering, cell-cell communication and vice versa [42]. Herein, the protein folding of the designed vaccine construct was evaluated through all-atoms molecular dynamics simulation. Using AMBER20 [43,44], the structure was neutralized and solvated with TIP3PBOX of 14.0 Å. To relax the structure and remove bad contacts, a two step gentle minimization of 6000 and 3000 steps was performed. The system was then heated and equilibrated. MD was run for 100 ns to evaluate the structural-dynamic properties of the vaccine ensemble. For protein stability, folding and flexibility CPPTRAJ and PTRAJ packages embedded in AMBER20 were used [45].

### 2.3. Evaluation of the Immune Response Triggering Properties of MEVC

#### 2.3.1. Molecular Docking of the Vaccine

The vaccine was docked with TLR4 (PDB ID:3FXI) using the PatchDock [46] server to disclose the binding orientation and interactions of the vaccine with TLR4. The docking was run by PatchDock, involving the separation of a molecule’s surface into small but proper shape patches. These patches provide a basis for complex patterning and can be visualized as puzzle pieces. Shape matching algorithms were then used to identify the best fixing patches for predicting the best possible, stable orientation of one molecule to another. Moreover, Fast Interaction Refinement in Molecular Docking (FireDock) [47] was applied to the solutions of PatchDock to guide the selection of the complex with the lowest global binding energy. The global energy can be split into the following components: atomic contact energy, van der Waals interaction energy, both attractive and repulsive, and hydrogen bonding energy.

#### 2.3.2. Binding Free Energy Calculation

The binding free energy of the MEVC and TLR4 was estimated by using the MM-GBSA approach. The HawkDock server calculates the vdW, electrostatic energy, generalized born, and the total binding free energy of a protein-protein complex by using the MMPBSA.py package of AMBER simulation package [48].

#### 2.3.3. In Silico Cloning and Expression

For maximum expression in the host cell, codon optimization was performed. Herein, *Escherichia coli* K12 was considered as an expression host. To optimize the sequence, Jcat tool (Java Codon Adaptation Tool) [49] was utilized to reverse translate the given protein sequence and obtain an optimized nucleotide sequence with suitable restriction sites. For in silico expression, pET28a (+) plasmid was selected, and snap gene software was used to perform the expression [50].

#### 2.3.4. Immune Simulation

Using immune simulation, the interaction between the foreign particles and the immune system may be projected. To this purpose, an online C-ImmSim server was used. As an agent-based modeling approach, this server is used to comprehend the host immune system’s dynamics in response to a foreign particle [51]. The relevance of learning via organism-environment interactions is emphasized in the agent-based concept. This method is part of a current trend in computational learning and development models that focuses on researching autonomous organisms in virtual or actual environments. Using the PSSM model, the server measures the reaction of the immune system against the antigen. Production of various immune substances such as antibodies, interferon, and cytokines production upon vaccine administration is estimated. Additionally, Th1 (T helper cell 01) and Th2 (T helper cell 02) responses are also predicted by the webserver. The server plotted Simpson Index or D (a measure of diversity) at default parameters.

## 3. Results

### 3.1. Sequence Retrieval and Antigenicity Profiling

The proteome of the tick-borne virus was fetched from the NCBI database and scanned for potential humoral immunity mediating B cell and cellular immunity mediating T cell epitopes, to subsequently be used in the design of a multi-epitope peptide vaccine ensemble. Figure 1 represents the overall flow of the work. The whole polyprotein, which includes three structural and seven non-structural proteins, were subjected to a vaccine designing pipeline.

### 3.2. Unveiling Cytotoxic T Lymphocytes (CTL) Epitopes

From the whole proteome of the tick-borne virus, a total of 31 peptides each 9-mer in length were predicted as potential CTL epitopes, as tabulated in Table 1. Further, filtration based on good binding affinity score and efficient antigenic nature showed only nine epitopes were considered as final CTL epitopes. The final selection of CTL epitopes was based on the highest combined score. These nine epitopes were then added to the final multi-epitopes’ vaccine construct.

### 3.3. Prioritizing Helper T Lymphocytes (HTL) Epitopes

While predicting HTL epitopes, five human alleles, namely HLA- DRB1*01:02, HLA-DRB1*01:01, HLA-DRB1*01:04, HLA-DRB1*01:05, and HLA DRB1*01:03, were considered as to which virus sequence peptides bind with high efficiency. Epitopes with the lowest percentile rank were selected for the final multi-epitope vaccine construct. Previously, epitopes with lowest percentile ranks were also reported to possess strong antigenic properties. The selected HTL epitopes include: 73–87 (HLA-DRB1*07:01), 76–90 (HLA-DRB1*07:01), 65–79 (HLA-DRB5*01:01), 63–77 (HLA-DRB5*01:01), 35–49 (HLA-DRB1*15:01), 33–47 (HLA-DRB1*15:01) and 36–50 (HLA-DRB1*15:01), as presented in Table 2. Both CTL and HTL epitopes were ranked on antigenic and high binding affinity for the MHC alleles.

### 3.4. Prediction of B Cell Epitopes

**Figure 2:**
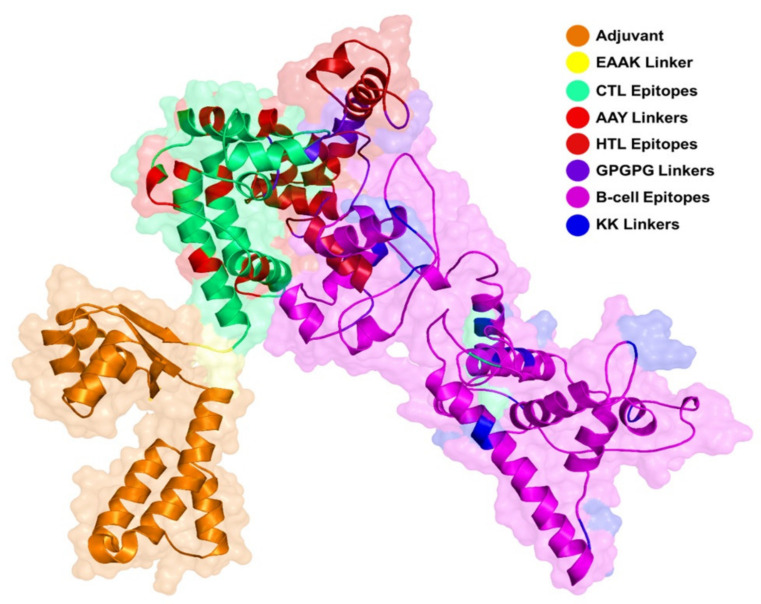
Showing each component of the final vaccine. Adjuvant, linkers, CTL, HTL, and B-cell epitopes are colored differently.

B cell epitopes are key mediators in provoking the humoral immune response and long-term immunity. Prediction of B cell epitopes with the highest scores are favorable for MEVC. The higher the score, the most potential the epitope. Thus, epitopes of 20-mer in length and scores >0.9 were selected for the MEVC. Table 3 shortlists the ten epitopes with the highest score. The ten epitopes with a score greater than default and length 20-mer were selected for downward analysis. Conformational B-cell epitopes were predicted through ElliPro. The finally selected B-cell epitopes are given in Figure 2. The score for these predicted B cell epitopes ranged from 1 to 0.99.

### 3.5. Multi-Epitope Peptide Vaccine Design

**Figure 3:**
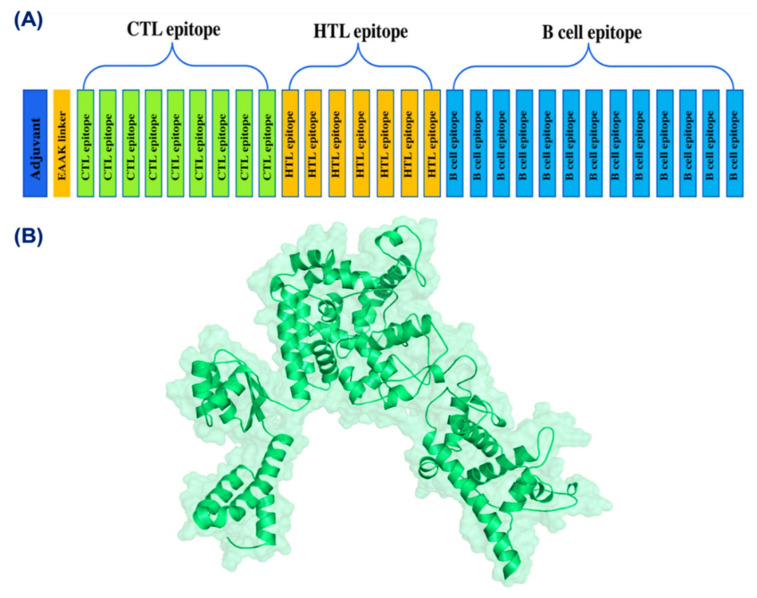
(**A**) Schematic presentation of the final multi-epitope vaccine. Nine CTL, seven HTL, and thirteen B-cell epitopes were fused together using EAAK, AAY, GPGPG, and KK linkers. Adjuvant was also added to increase the stability and folding of the construct. (**B**) 3D structure of the final vaccine construct.

The selected 9 CTL epitopes, 7 HTL epitopes, and 13 B-cell epitopes were fused with each other using different linkers. For CTL epitopes, AAY linkers were used, whereas GPGPG linkers were employed for linking HTL epitopes. Additional joining of B cell epitopes to the T cell epitopes peptide was performed through the KK linker. The total length of the final epitope was 665 amino acids. Schematically, the final ensemble vaccine structure is provided in Figure 3.

### 3.6. Prediction of Vaccine 3D Structure and Validation

For the vaccine construct, five 3D models were produced from its amino acid sequence by the Robetta ab initio modeling server. The models were assessed using the Ramachandran plot, in addition to ProSA-web and ERRAT. The selected 3D model of the vaccine was then subjected to a structure assessment phase. ProCheck for protein model showed that 91.9% of amino acids were plotted in the most favorable areas, 7.7% in allowed areas, and only 0.4% in disallowed areas.

**Figure 4:**
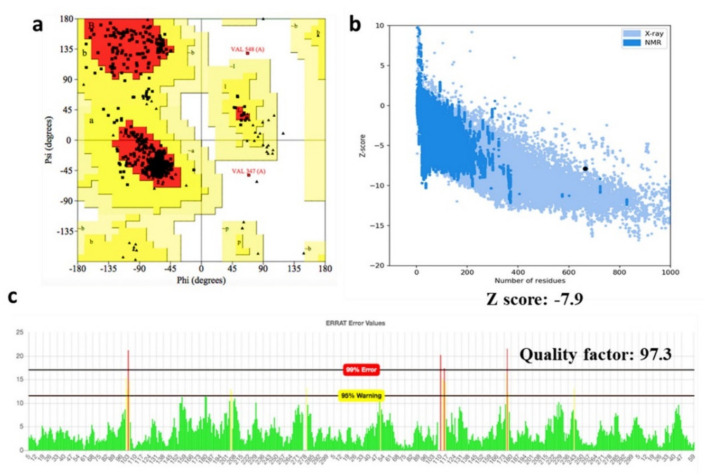
Vaccine 3D structure modeling and validation. (**a**) Ramachandran plot of the predicted 3D structure (**b**) PROSA Z-score of the vaccine, (**c**) ERRAT validation for the predicted structure.

Similarly, ERRAT and ProSA-web calculated and verified the overall quality of the basic 3D model. The resulted overall quality factor reported by ERRAT was 97.3%, and the ProSA-web showed a score of −7.9 for the 3D model of the vaccine construct, which proved the accuracy of the model. Results from ProSA-web and rampage are given in Figure 4. All the mentioned analyses found model 2 as the most optimal model for the vaccine hence considered in further analyses.

### 3.7. D Structure Validation

The best model initiated by the Robetta server, that was selected on the basis of certain factors such as RMSD value and high GDT-HA score, was further used for 3D structure analysis. Moreover, ProCheck results for the vaccine construct showed 90.6% amino acids in the favored region, and 9.4% amino acid, were plotted in allowed regions (Figure 4A). ProSA–web and ERRAT validated the three-dimensional structure of the vaccine construct with a quality factor of 94.1%, and Z-score −6.48, by ERRAT and ProSA-web, respectively (Figure 4C,D).

### 3.8. Prediction of Allergenicity

Allergenicity of the multi-epitope vaccine was examined, and demonstrated to harbor no sequence responsible for allergenic reactions. The E-value of the vaccine was <threshold 0.001 value. The nearest protein was UniProtKB accession number Q8IWY9.

### 3.9. Antigenicity of the Vaccine Construct

The antigenicity score for the final vaccine construct was predicted to be 0.5, which was greater than the threshold of 0.4. This demonstrates the vaccine possesses significant antigenic properties and is capable of provoking strong immune responses.

### 3.10. Physiochemical Properties of the Designed Vaccine

The molecular weight of the vaccine was 71.3 kDa and thus can be purified easily in follow up wet-lab experimentation. The theoretical protrusion index (PI) of the vaccine was 9.66, referring to its basic nature. In vivo half-life of the vaccine in *E. coli* was >10 h. The instability value was determined as stable, as it had a predicated instability score of 25.67 (<40 is categorized as stable). The aliphatic index score was 75.17, which means the vaccine thermostable nature at different temperatures. The Grand Average of Hydropathy value reported for the vaccine was −0.35, making it a hydrophilic molecule.

### 3.11. Prediction of Secondary Structure Elements

**Figure 5:**
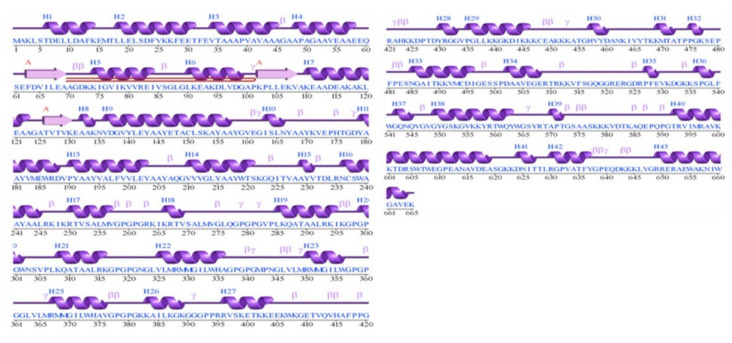
Graphical depiction of the vaccine secondary structure. Each secondary structure element is represented by a different symbol given above each region.

The secondary structure elements of the vaccine are comprised of coils (39.88%), strand (29.91%), helix (28.04%), and beta-turn (2.18%). Graphically, the secondary structure of the vaccine is given in Figure 5.

### 3.12. Protein Folding Evaluation through All-Atoms MD Simulation

**Figure 6:**
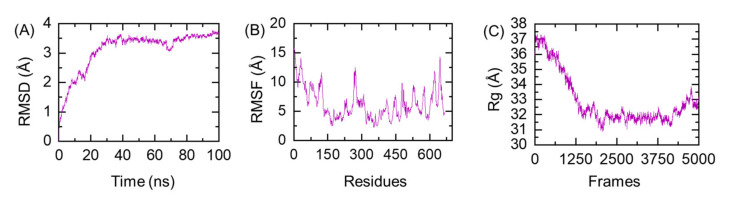
Stability, compactness and flexibility evaluation of the MEVC. (**A**) shows the RMSD, (**B**) represents the compactness (Rg), while (**C**) shows the residual flexibility as RMSF.

Prior to validation of the vaccine ensemble and its interaction with the immune receptor, the folding of the designed MEVC was evaluated. MD simulation-based stability and folding is of great interest to reveal different dynamic properties such as the function of a loop in a protein, importance of different residues in interaction with different proteins, the impact of protein-protein interfaces, and mutations [52,53]. Herein, the stable folding of the MEVC was revealed through root mean square deviation (RMSD) as a function of time. As given in Figure 6A, the folding of the MEVC was very stable and did not deviate from the mean structure significantly. The system reached the equilibrium position at 20 ns, and for the rest of the simulation time the folding pattern remained uniform.

This shows that the structural folding was already stabilized at 20 ns, and revealed that MEVC had proper stable folding. Similarly, the compactness of the protein evaluated as Rg (radius of gyration), Figure 6B, also showed that the structural compactness remained high during the first 20 ns and gradually decreased over the simulation time, and soon after reaching 40 ns the Rg difference between the initial structure and the structure at 40 ns was ~6.0 Å, explaining that the protein folding reached an equilibrium point, and this pattern remained consistent until 100 ns. Moreover, the residual flexibility remained less than 1.5 Å, which shows that the uniformly dispersed helices and beta-sheets have stabilized the structural folding. The RMSF (root mean square fluctuation) of the MEVC is shown in Figure 6C. Conclusively, this shows that the protein behavior in a dynamic environment revealed stabilized folding. For instance, the results of an all-atoms simulation and experimental protein evaluation were reported to be greatly uniform [52,54].

### 3.13. Protein-Peptide Docking of the Vaccine to TLR4

**Figure 7:**
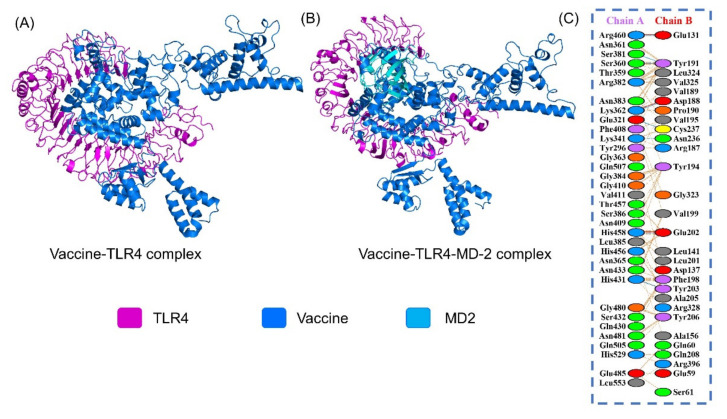
(**A**) Vaccine-TLR4 docked complex. (**B**) Vaccine-TLR4-MD2 docked complex. The receptor TLR4 is colored as magenta to contrast the blue color vaccine. The MD2 is shown as cyan in panel (**B**). The complex is also shown as a sphere. (**C**) Interacting residues are also given as right panel.

The PatchDock server generated 100 docked solutions of the vaccine with respect to the TLR receptor and ranked as per geometry and electrostatic interdependence of the receptor surface. Refinement in FireDock re-scored the docked solutions and ordered them on the basis of global binding energy (KJ/mol). Additionally, graphical information of intermolecular interactions was elucidated via the online PDBsum and PDBePISA databases. Chemically, 8 hydrogen bonds were found between the vaccine (Chain A) and TLR4 (Chain B); 460–131, 381–191, 360–191, 262–188, 321–237, 341–236, 458–202, and 431–203. Additionally, 414 non-bonded interactions were revealed in the formation of vaccine and TLR4 complex (Figure 7A). Moreover, we also performed the docking of MEVC with TLR4 in complex with MD2 as shown in Figure 7B. The data show that the binding of the MEVC was conserved even if MD2 was available. The MEVC occupied the same site and blocked the same residues as reported in the TLR4-MEVC complex alone. This shows that the designed MEVC accessibility of the binding site is highly accurate.

### 3.14. Binding Free Energy Evaluation of MEVC-TLR4

Binding free energy is a widely practiced approach to reveal the binding affinity of protein-protein, protein-ligand, and protein-DNA/RNA complexes accurately [55,56,57,58,59]. The binding energy of the MEVC-TLR4 was estimated using MM-PBSA.py script. The results revealed that the complex had the total binding energy of −87.50 kcal/mol, while the other contributing factors, such as vdW, electrostatic and SA, were reported to be −168.72 kcal/mol, −4858.53 kcal/mol and 4958.08, respectively. This shows a more robust interaction of the MEVC with the host immune receptor TLR4.

### 3.15. Codon Optimization and In Silico Cloning

**Figure 8:**
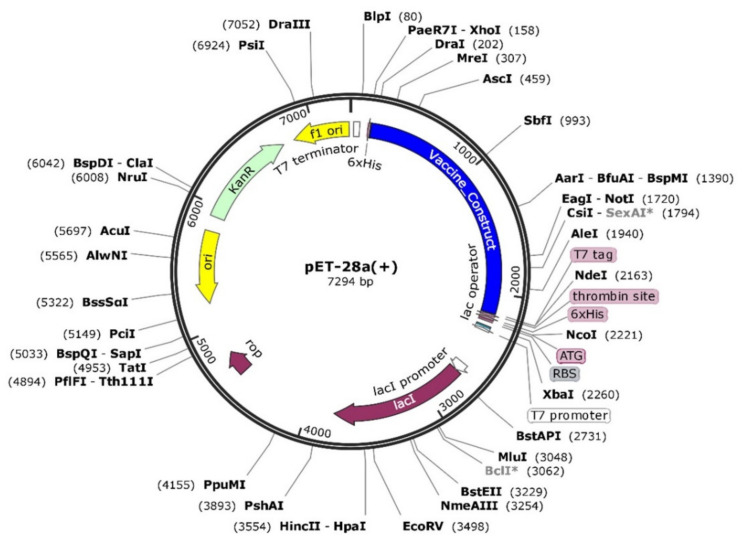
In silico cloning of the vaccine (shown in blue) into the pET28a (+) vector (shown by red color). The calculated CAI value 0.93 and the GC contents 49% shown after codon optimization by JCat server.

The vaccine sequence codon usage was adjusted according to *E. coli* (strain K12) system to achieve its high expression (Figure 8). The sequence length of the optimized codons sequence was 1998 nucleotides. The vaccine had a Codon Adaptation Index (CAI) score of 0.93, with a GC content of 49% (optimum range is between 30% and 70%), both pointing to the vaccine’s high expression in *E. coli* cell. Afterwards, the optimized sequence of the vaccine was cloned into the pET28a (+) vector.

### 3.16. Immune Simulation Analysis

**Figure 9:**
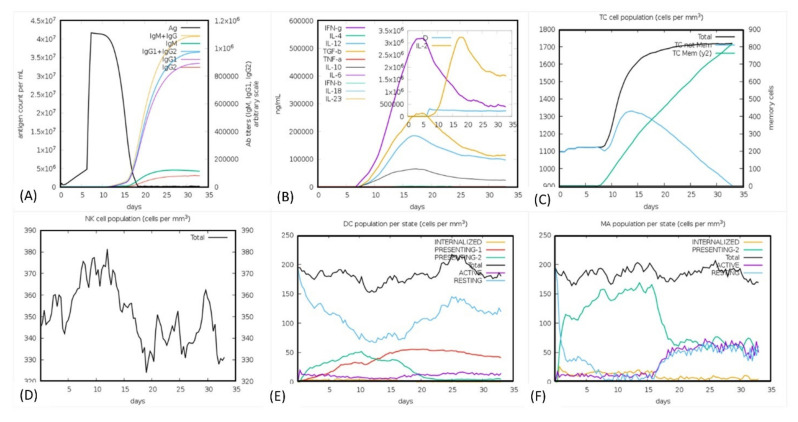
(**A**) Antibodies titer, (**B**) cytokines and interleukins concentrations, (**C**) T cell population, (**D**) NK cell population (**E**) dendritic cell population, and (**F**) macrophage population production in response to the antigen.

The human immune system response was monitored through in silico immune simulation upon the injection of the antigen in several doses. It can be seen (Figure 9A) that the immune response was significantly triggered, and the antibody titer was very high after the injection. It can clearly be observed that the combined IgM + IgG titer remained 4 × 10^7^ xx/mL. The IgM alone titer was reported to be around 3.5 × 10^7^ xx/mL, while Ig1 was reported as 3.2 × 10^7^ xx/mL. The graph shows that the antigen titer was initially lower until the 5th day after the injection, then the titer increased, however after five days the titer gradually decreased and the IgM + IgG, Ig1 production was increased. At the 13th day the total neutralization of the antigen was observed with the production of other immune factors. We also checked the interleukin (IL) and cytokines responses. As presented in Figure 9B the IFN-g and IL-2 titer were significantly high. This shows the consistent and robust immune triggering response upon the injection. The cellular immune system response to pathogen identification at the re-encounter was also very strong, including the development of memory cells. The population of the T cell was reported to >1700 cells/mm^3^. The maximum concentration of 380 cells/mm^3^ for the phagocytic natural killer cell population was reported, while the dendritic cells and phagocytic macrophage population were reported to be 200 cell/mm^3,^ respectively. In the case of the TLR4 response, the production of TNF-alpha in higher titer is associated with lipopolysaccharides (LPS) designed vaccine which are bacterial and absent in viruses [60], implying that the production of IL-6 in higher titer works in the neutralization of viral antigen. Hence these results confirm that our vaccine candidate effectively triggered the immune response upon the injection. For instance, negative control results show that the antigen titer was given as high as 3.1 × 10^2^ but still the response could not be provoked efficiently. The antigen remained in higher load for the first 20 days and different injections. This shows that the positive MEVC triggered a more robust immune response than the negative construct. The results of immune server are shown in Figure 9A–F.

In summary, the current study uses computational modeling approaches to design MEVC for tick-borne virus. This multi-steps methodology followed a conventional pipeline initiated by retrieving the key targets and the prediction of antigenic and allergenic properties. In the second step CTL, HTL and B cell epitopes were predicted and shortlisted based on combined score, percentile rank and total score. Next, a multi-epitopes vaccine was constructed based on the selected epitopes joining with linkers. Adjuvant was also added for immunogenic properties augmentation. In the next steps, evaluation of the MEVC was carried out using molecular docking, immune simulation, MD simulation, binding free energy calculations, in silico cloning and expression. The final vaccine candidate was reported to be antigenic and non-allergenic, and potentially triggers the host immune response.

## 4. Discussion

Infectious agents spread by tick bites are sparked by tick-borne pathogens that afflict humans and other species. Infection of a host of diseases, including rickettsia and other forms of bacteria, viruses, and protozoa, cause them. Since more than one disease-causing agent can be identified in human ticks, patients can be afflicted with more than one virus at the same time, exacerbating the complexity of diagnosis and treatment [61]. Factors that facilitate their survival in infection are required to be identified for drugs and vaccine designing. Demonstration of surface-visible antigenic epitopes is necessary for successful control and full eradication of this virus. This should be the primary target for effective vaccines’ potential production [62]. In the efficient prevention of infectious diseases, vaccination plays an integral role [63]. Multi-epitope-based peptide vaccines are advantageous because, relative to traditional vaccines, they are viable, extremely safe, non-toxic, and simple in the engineering process, which may have several issues in immune susceptible people [64,65]. These peptides-based therapeutic vaccines designed from the proteome of a pathogen are highly targeted and immunogenic [23,66]. In addition, such vaccines have the potential to evoke both cell mediated and humoral immunity. A number of multi-epitopes-based vaccines have been designed against different pathologies including cholera, leishmaniasis, cancers and dengue, and their benefits have been further revealed in Schistosoma*, Helicobacter,* and cancer [18,20,67,68,69,70]. Immunization is the most efficient and secure tool for the prevention of infectious diseases and public health. The advancement and development of multi-epitope subunit vaccines have increased the opportunity to find new antigenic targets by applying immunoinformatics approaches, due to the rapid expansion and accessibility of enormous proteomics results [20]. As the prevalence of tick-borne diseases grows and the regional areas in which they are found growing, the complex, and sometimes overlapping, clinical manifestations of these diseases must continually be differentiated by health professionals. The tick-human spread of illness has caused many high-profile deaths, including the death at the age of 66 of former Senator Kay Hagan in the United States in 2019 [71]. These methods were widely applied by different studies to design a multi-epitopes vaccine against bacterial and viral pathogens [18,63,67,72,73]. Herein, we have also used computational methods to mine the tick-borne virus proteome to design a multi-epitopes subunit vaccine.

Our analysis showed that there were ten proteins, of which three were structural, while seven were non-structural. Our analysis revealed that the polyprotein possessed antigenic properties and was the best option for vaccine design. Our analysis predicted 9 CTL epitopes, 7 HTL epitopes, and 13 B-cells as the most potential antigenic peptides considered for the final vaccine construct. The final vaccine construct using adjuvant and linkers to join these epitopes was used, and the vaccine candidate was classified as non-allergenic and strong antigenic. Furthermore, the physicochemical properties also revealed that the candidate vaccine properties were acceptable. The docking and in silico cloning further confirmed its interaction with TLR-4 and its expression in the *E. coli* host for downstream processing. Any mutation in the protein sequence may not potentially affect the efficacy of this vaccine for different variants of tick-borne, if they emerged, because the combination of different antigenic epitopes from different proteins preserves the antigenic properties and could not be significantly affected because of the multiple proteins’ epitopes combination. This is unlike the current vaccines for SARS-CoV-2 which are either entirely based on receptor-binding domain (RBD) or Spike protein.

Furthermore, immune simulation revealed that the vaccine candidate efficiently triggered the immune response, and the host immune factors were released in higher titer. Thus, these results suggest that our proposed vaccine candidate has the potential to be tested in clinical trials at the earliest opportunity. Our vaccine construct’s safety, efficacy, and other properties make it a suitable choice for in vitro and in vivo analysis.

Conclusively, in response to the recent outbreak of tick-borne disease, we have attempted to design a multi-epitopes vaccine using computational modeling approaches. Our vaccine candidate efficiently triggered the immune response using a computational algorithm for immune response prediction. We suggest the clinical testing of this vaccine candidate for its therapeutic potential both in vitro and in vivo to fight against tick-borne disease-causing pathogens.

## 5. Conclusions

In conclusion, this study provides a deep insight into the structural basis for vaccine development against tick-borne viruses. The designed multi-epitopes subunit vaccine could trigger robust immune response and hence demands the in vitro and in vivo investigation for the eradication of tick diseases.

## Figures and Tables

**Table 1:** Predicted CTL epitopes. The selection of appropriate candidates was based on C-terminal cleavage, TAP scores, and antigenicity. The CTL epitopes shown in bold are the finally selected epitopes for multi-epitopes vaccine construct.

Residue No.	Peptide Sequence	MHC Binding Affinity	Rescale Binding Affinity	C-Terminal Cleavage Affinity	Transport Affinity	Prediction Score	MHC-I Binding	Antigenecity Score
2237	SSDDNKLAY	0.7693	3.2662	0.9150	2.9740	3.5521	YES	Non-antigenic
**184**	**NVDGVYLEY**	**0.6968**	**2.9587**	**0.9788**	**3.0560**	**3.2583**	**YES**	**0.876**
3035	STLNGGLFY	0.6183	2.6251	0.9456	3.0220	2.9180	YES	Non-antigenic
98	ATDWMSWLL	0.5851	2.4842	0.9229	0.9550	2.6704	YES	Non-antigenic
**1788**	**SIAARGHLY**	**0.4418**	**1.8759**	**0.9337**	**3.1000**	**2.1710**	**YES**	**0.524**
**3262**	**ETACLSKAY**	**0.4369**	**1.8550**	**0.2200**	**2.8480**	**2.0304**	**YES**	**0.691**
2130	CVSALDVFY	0.4011	1.7029	0.7949	3.1500	1.9797	YES	Non-antigenic
**3018**	**GVEGISLNY**	**0.3920**	**1.6642**	**0.9715**	**2.8940**	**1.9546**	**YES**	**1.4**
**451**	**KTILTMGEY**	**0.3915**	**1.6621**	**0.7760**	**2.9120**	**1.9241**	**YES**	**0.494**
**422**	**KVEPHTGDY**	**0.3583**	**1.5213**	**0.9717**	**3.0730**	**1.8207**	**YES**	**0.707**
971	HTDQSLWMR	0.3781	1.6055	0.7247	1.2050	1.7744	YES	Non-antigenic
3077	ATTIMQKAY	0.3578	1.5192	0.4362	3.0590	1.7375	YES	0.454
1955	RQDGRTDEY	0.3297	1.3998	0.9504	3.0380	1.6942	YES	Non-antigenic
800	EVSEWYDNY	0.3269	1.3879	0.9476	2.9870	1.6794	YES	Non-antigenic
2530	CTREEFFVY	0.3072	1.3045	0.9681	3.0520	1.6023	YES	0.617
1783	WTDPHSIAA	0.3502	1.4869	0.4722	0.7830	1.5185	YES	Non-antigenic
**2214**	**MAGVALIFY**	**0.2864**	**1.2161**	**0.5939**	**3.0020**	**1.4553**	**YES**	**0.532**
2855	MTDTTAFGQ	0.3395	1.4416	0.0703	0.1020	1.4470	YES	Non-antigenic
2813	RTWQYWGSY	0.2555	1.0848	0.9775	3.2670	1.3948	YES	Non-antigenic
**3342**	**VMEWRDVPY**	**0.2301**	**0.9770**	**0.9072**	**3.0960**	**1.2679**	**YES**	**1.79**
980	SMKNDTGTY	0.2236	0.9492	0.9495	3.2290	1.2530	YES	Non-antigenic
**1143**	**VALFVVLEY**	**0.2002**	**0.8501**	**0.9747**	**3.0660**	**1.1496**	**YES**	**0.652**
1235	VTTYFLLLV	0.2186	0.9279	0.7355	0.3550	1.0560	YES	Non-antigenic
**598**	**PTDSGHDTV**	**0.2370**	**1.0062**	**0.3920**	**0.2090**	**1.0545**	**YES**	**0.425**
553	VAHIEGTKY	0.1785	0.7579	0.9739	2.9240	1.0502	YES	Non-antigenic
2133	ALDVFYTLM	0.2087	0.8861	0.9760	0.3460	1.0498	YES	Non-antigenic
**1634**	**AQGVVVGLY**	**0.1806**	**0.7667**	**0.8269**	**3.1220**	**1.0469**	**YES**	**0.718**
**1677**	**WTSKGQITV**	**0.2126**	**0.9025**	**0.8831**	**0.2320**	**1.0466**	**YES**	**1.19**
448	SSEKTILTM	0.2101	0.8922	0.9417	0.2630	1.0466	YES	Non-antigenic
411	VYDANKIVY	0.1765	0.7494	0.8988	3.0520	1.0369	YES	Non-antigenic
**994**	**VTDLRNCSW**	**0.1987**	**0.8437**	**0.9180**	**0.7970**	**1.0212**	**YES**	**1.377**

**Table 2:** Predicted HTL epitopes. The table shows the specific allele against which the HTL epitopes are predicted. Furthermore, it also shows the methods and percentile rank of each peptide. The HTL epitopes shown in bold are the finally selected epitopes for multi-epitopes vaccine construct.

S. No.	Allele	Start	End	Peptide Sequence	Method	Percentile Rank
1	HLA-DRB1*07:01	73	87	AALRKIKRTVSALMV	Consensus (comb.lib./smm/nn)	0.03
2	HLA-DRB1*07:01	72	86	TAALRKIKRTVSALM	Consensus (comb.lib./smm/nn)	0.04
3	HLA-DRB1*07:01	74	88	ALRKIKRTVSALMVG	Consensus (comb.lib./smm/nn)	0.06
4	HLA-DRB1*07:01	75	89	LRKIKRTVSALMVGL	Consensus (comb.lib./smm/nn)	0.06
5	HLA-DRB1*07:01	76	90	RKIKRTVSALMVGLQ	Consensus (comb.lib./smm/nn)	0.06
6	HLA-DRB5*01:01	65	79	SVPLKQATAALRKIK	Consensus (smm/nn/sturniolo)	0.08
7	HLA-DRB5*01:01	66	80	VPLKQATAALRKIKR	Consensus (smm/nn/sturniolo)	0.08
8	HLA-DRB1*07:01	77	91	KIKRTVSALMVGLQK	Consensus (comb.lib./smm/nn)	0.13
9	HLA-DRB5*01:01	64	78	NSVPLKQATAALRKI	Consensus (smm/nn/sturniolo)	0.14
10	HLA-DRB5*01:01	63	77	WNSVPLKQATAALRK	Consensus (smm/nn/sturniolo)	0.43
11	HLA-DRB1*15:01	35	49	NGLVLMRMMGILWHA	Consensus (smm/nn/sturniolo)	0.72
12	HLA-DRB1*15:01	33	47	MPNGLVLMRMMGILW	Consensus (smm/nn/sturniolo)	0.73
13	HLA-DRB1*15:01	34	48	PNGLVLMRMMGILWH	Consensus (smm/nn/sturniolo)	0.73
14	HLA-DRB5*01:01	68	82	LKQATAALRKIKRTV	Consensus (smm/nn/sturniolo)	0.75
15	HLA-DRB5*01:01	67	81	PLKQATAALRKIKRT	Consensus (smm/nn/sturniolo)	0.81
16	HLA-DRB1*15:01	36	50	GLVLMRMMGILWHAV	Consensus (smm/nn/sturniolo)	0.88
17	HLA-DRB1*15:01	37	51	LVLMRMMGILWHAVA	Consensus (smm/nn/sturniolo)	0.93

**Table 3:** Predicted linear B-cell epitopes for the virus polypeptide. The predicted score for each peptide is given against each B-Cell epitope.

S. No	Position	Epitope	Score
1	3	KKAILKGKGGGPPRRVSKET	1
2	1578	EEKWKGETVQVHAFPPGRAH	0.999
3	870	DPTDYRGGVPGLLKKGKDIK	0.999
4	401	CEAKKKATGHVYDANKIVYT	0.999
5	1809	MTATPPGKSEPFPESNGAIT	0.999
6	2654	VMCDIGESSPDAAVEGERTR	0.998
7	1493	VFSGQGGRERGDRPFEVKDG	0.995
8	1518	SPGLFWGQNQVGVGYGSKGV	0.995
9	2812	YRTWQYWGSYRTAPTGSAAS	0.995
10	2870	KVDTKAQEPQPGTRVIMRAV	0.995
11	2049	TDRSWTWEGPEANAVDEASG	0.993
12	1987	DNITTLRGPVATFYGPEQDK	0.993
13	3362	LVGRRERAEWAKNIWGAVEK	0.99

## Data Availability

All data will be provided on reasonable demand.
