# Correction: Suleman et al. Immunoinformatics and Immunogenetics-Based Design of Immunogenic Peptides Vaccine against the Emerging Tick-Borne Encephalitis Virus (TBEV) and Its Validation through In Silico Cloning and Immune Simulation. *Vaccines* 2021, *9*, 1210

**DOI:** 10.3390/vaccines12080920

**Published:** 2024-08-15

**Authors:** Muhammad Suleman, Muhammad Tahir ul Qamar, Kiran, Samreen Rasool, Aneela Rasool, Aqel Albutti, Noorah Alsowayeh, Ameen S. S. Alwashmi, Mohammad Abdullah Aljasir, Sajjad Ahmad, Zahid Hussain, Muhammad Rizwan, Syed Shujait Ali, Abbas Khan, Dong-Qing Wei

**Affiliations:** 1 Centre for Biotechnology and Microbiology, University of Swat, Swat 19200, Pakistan; suleman@uswat.edu.pk (M.S.); zahid@uswat.edu.pk (Z.H.); m.rizwan@uswat.edu.pk (M.R.); shujaitswati@uswat.edu.pk (S.S.A.); 2 College of Life Science and Technology, Guangxi University, Nanning 530004, China; m.tahirulqamar@hotmail.com; 3 Department of Plant Breeding and Genetics, University of Agriculture, Faisalabad 38000, Pakistan; kiranzahra9999@gmail.com; 4 Department of Biochemistry, Government College University, Lahore 54000, Pakistan; samaruaf@yahoo.com; 5 Department of Botany, University of Okara, Okara 56300, Pakistan; aneelarasool3@gmail.com; 6 Department of Medical Biotechnology, College of Applied Medical Sciences, Qassim University, Buraydah 51452, Saudi Arabia; 7 Department of Biology, College of Education, Majmaah University, Al Majma’ah 15341, Saudi Arabia; n.alsowayeh@mu.edu.sa; 8 Department of Medical Laboratories, College of Applied Medical Sciences, Qassim University, Buraydah 51452, Saudi Arabia; aswshmy@qu.edu.sa (A.S.S.A.); mjasr@qu.edu.sa (M.A.A.); 9 Department of Health and Biological Sciences, Abasyn University, Peshawar 25120, Pakistan; sahmad@bs.qau.edu.pk; 10 Department of Bioinformatics and Biological Statistics, School of Life Sciences and Biotechnology, Shanghai Jiao Tong University, Shanghai 200240, China; abbaskhan@sjtu.edu.cn; 11 State Key Laboratory of Microbial Metabolism, Shanghai-Islamabad-Belgrade Joint Innovation Center on Antibacterial Resistances, Joint Laboratory of International Cooperation in Metabolic and Developmental Sciences, Ministry of Education and School of Life Sciences and Biotechnology, Shanghai Jiao Tong University, Shanghai 200030, China; 12 Peng Cheng Laboratory, Vanke Cloud City Phase I Building 8, Xili Street, Nashan District, Shenzhen 518055, China

The authors would like to make the following correction to this published paper [1].

After publication of the paper [1], it was found that the abstract of the paper is an exact duplication of the abstract from a previously published paper [2] by the same authors. The error was due to a wrong version being mistakenly uploaded by the authors during the process. The corrected abstract is listed below:

**Abstract:** Tick-borne encephalitis virus (TBEV), belonging to the Flaviviridae family, is transmitted to humans via infected tick bites, leading to serious neurological complications and, in some cases, death. The available vaccines against the TBEV are reported to have low immunogenicity and are associated with adverse effects like swelling, redness and fever. Moreover, these vaccines are whole-organism-based, carry a risk of reactivation and potential for significant mortality. Consequently, to design a potential antigenic and non-allergenic multi-epitope subunit vaccine against the TBEV, we used an immunoinformatic approach to screen the Tick-borne virus proteome for highly antigenic CTL, HTL and B cell epitopes. The proper folding of the constructed vaccine was validated by a molecular dynamic simulation. Additionally, the molecular docking and binding free energy (−87.50 kcal/mol) further confirmed the strong binding affinity of the constructed vaccine with TLR-4. The vaccine exhibited a CAI value of 0.93 and a GC content of 49%, showing a high expression capability in *E coli*. Moreover, the analysis of immune simulation demonstrated robust immune responses against the injected vaccine and clearance of the antigen with time. In conclusion, our vaccine candidate shows promise for both in vitro and in vivo analyses due to its high immunogenicity, non-allergenicity and stable interaction with the human TLR-4 receptor.

The authors state that there are no issues except the abstract after checking the manuscript, all the data, the sequences, the targets, the analysis and the results in detail. The scientific conclusions are unaffected. The authors apologize for this mistake. The original article has been updated.
